# Laparoscopic ureterolithotomy and retrograde flexible ureteroscopy-assisted transperitoneal laparoscopic ureteroureterostomy for a huge ureteropelvic junction stone and multiple small renal stones: A CARE-compliant case report

**DOI:** 10.1097/MD.0000000000026655

**Published:** 2021-07-16

**Authors:** Sheng-Feng Chou, Po-Fan Hsieh, Wei-Ching Lin, Chi-Ping Huang

**Affiliations:** a Department of Urology, China Medical University Hospital, Taichung, Taiwan; b School of Medicine, China Medical University, Taichung, Taiwan; c Graduate Institute of Biomedical Sciences, School of Medicine, China Medical University, Taichung, Taiwan; d Department of Radiology, China Medical University Hospital, Taichung, Taiwan.

**Keywords:** case report, laparoscope-assisted retrograde intrarenal surgery, laparoscopic ureterolithotomy, retrograde intrarenal surgery

## Abstract

**Rationale:**

Concurrent kidney and ureteral stones are always complicated and a clinical challenge. Improvements in endoscopic equipment have led to the widespread adoption of retrograde intrarenal surgery, which has a good stone clearance rate. On the other hand, laparoscopic ureterolithotomy (LUL) has been reported to be non-inferior to retrograde flexible ureteroscopy in stone-free rate and the need for axillary procedures, and to have a significantly lower rate of post-operative sepsis compared to retrograde flexible ureteroscopy. We describe a case managed with LUL followed by laparoscope-assisted retrograde intrarenal surgery (LA-RIRS) in a single operation for a large upper ureteral stone and small renal stones, which is usually challenging and requires axillary procedures.

**Patient concerns:**

The patient was a 66-year-old male with underlying hypertension and diabetes mellitus. He reported severe flank pain after receiving endoscopic management of concurrent right ureteropelvic junction stone and multiple renal stones about 1 month previously.

**Diagnosis:**

The residual stones were reassessed using non-contrast computed tomography before surgery. A 2.8-cm residual ureteropelvic junction stone and multiple renal stones with a maximum length of 1 cm were found. A second operation was considered to be necessary due to the deterioration of his renal function and refractory flank pain.

**Interventions:**

We performed LUL followed by LA-RIRS. Two surgeries were completed in a single operation. The Jackson–Pratt drain was removed 3 days after the operation.

**Outcomes:**

After the surgery, no high-grade complications were recorded according to the Clavien Dindo classification. A follow-up kidney, ureter, and bladder radiograph performed 2 months after the operation revealed no residual stones. Renal echo revealed no obstructive nephropathy 1 month after double-J catheter removal.

**Conclusion:**

LUL with LA-RIRS with a stone basket for renal stone extraction is a safe and feasible technique, and no step surgery or axillary procedures were needed in our case. If clinical cases with a huge stone burden over the ureter are indicated for LUL with concurrent small renal stones, LUL with LA-RIRS can be an alternative option.

## 1 Introduction

Concurrent kidney and ureteral stones have increasingly been managed with endoscopic methods in recent years due to the development of newer generation lithotripters and semi-rigid ureteroscopy along with holmium laser lithotripsy.^[1,2]^ Urolithiasis can often be managed with a minimally invasive approach, with a similar stone-free rate and safety profile to traditional shock wave lithotripsy (SWL) and percutaneous nephrolithotomy (PCNL).^[3–6]^ However, the management of concurrent kidney and ureteral stones can be time-consuming when using an endoscopic method, and it is associated with a high risk of sepsis.^[7]^ In addition, the flexibility of a flexible ureteroscope is limited in a renal pelvis with high-grade hydronephrosis, which then affects the operation field and stone-free rate.^[8,9]^ On the other hand, laparoscopic ureterolithotomy (LUL) has been shown to be non-inferior to retrograde flexible ureteroscopy (fURS) in stone-free rate and the need for axillary procedures, and to have a significantly lower rate of post-operative sepsis with huge ureteral stones.^[10–13]^ In this article, we report the case of a 66-year-old male patient who had previously undergone endoscopic management with double-J catheter (D-J) stenting but still had a heavy stone burden in the ureteropelvic junction (UPJ) and lower pole renal calyx. We conducted LUL with laparoscope-assisted retrograde intrarenal surgery (LA-RIRS) for residual stones and achieved excellent results.

## 2 Case presentation

**Figure 1:**
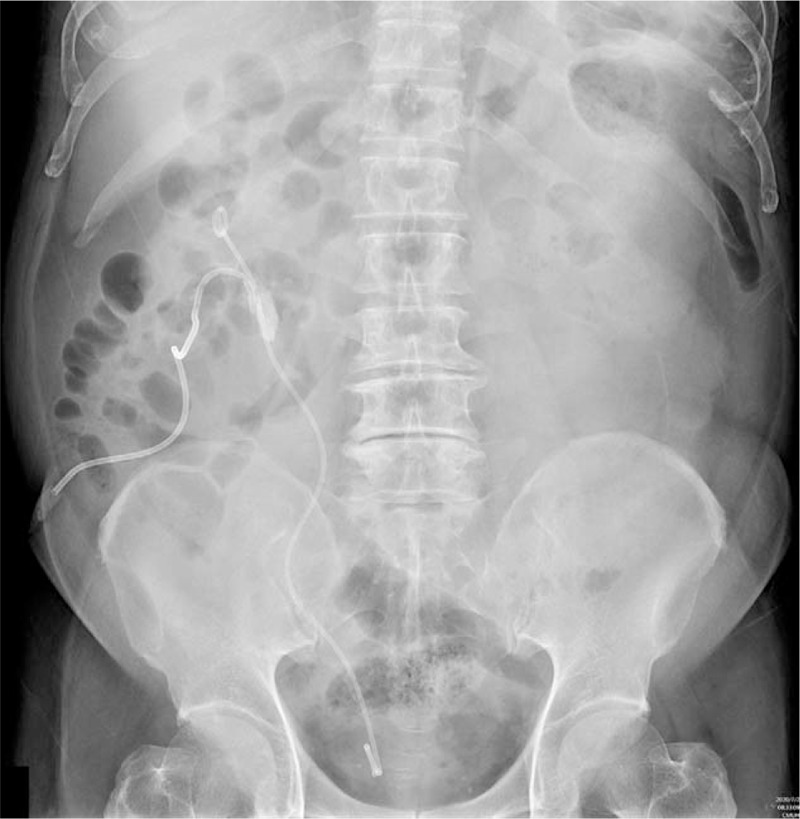
Preoperative findings of a kidney, ureter, and bladder radiograph that were suggestive of a large ureteropelvic junction stone about 2.8 cm in size and multiple renal stones with a maximum size of about 1 cm with D-J stent and percutaneous nephrostomy. D-J = double-J catheter.

A 66-year-old male patient presented with a history of hypertension, diabetes mellitus, and concurrent renal and ureteral stones after receiving endoscopic management at another hospital about 1 month previously. Residual stones in the UPJ and lower calyx were noted in a kidney, ureter, and bladder radiograph (Fig. 1) after the previous operation. The patient reported severe flank pain, and a second operation was considered to be necessary due to the deterioration of his renal function. According to the records of the previous operation, a tight connection between the UPJ stones and adjacent ureter mucosa made it difficult to push back to the renal pelvis, and the severe angulation made it difficult to reach the edge of the UPJ stones. Therefore, ureteroscopic lithotripsy had been performed for the distal stones with PCNL for the renal pelvis stones in the previous operation. Consequently, a residual UPJ stone about 2.8 cm in size and multiple small lower calyx stones were left with D-J stenting and percutaneous nephrostomy.

**Figure 2:**
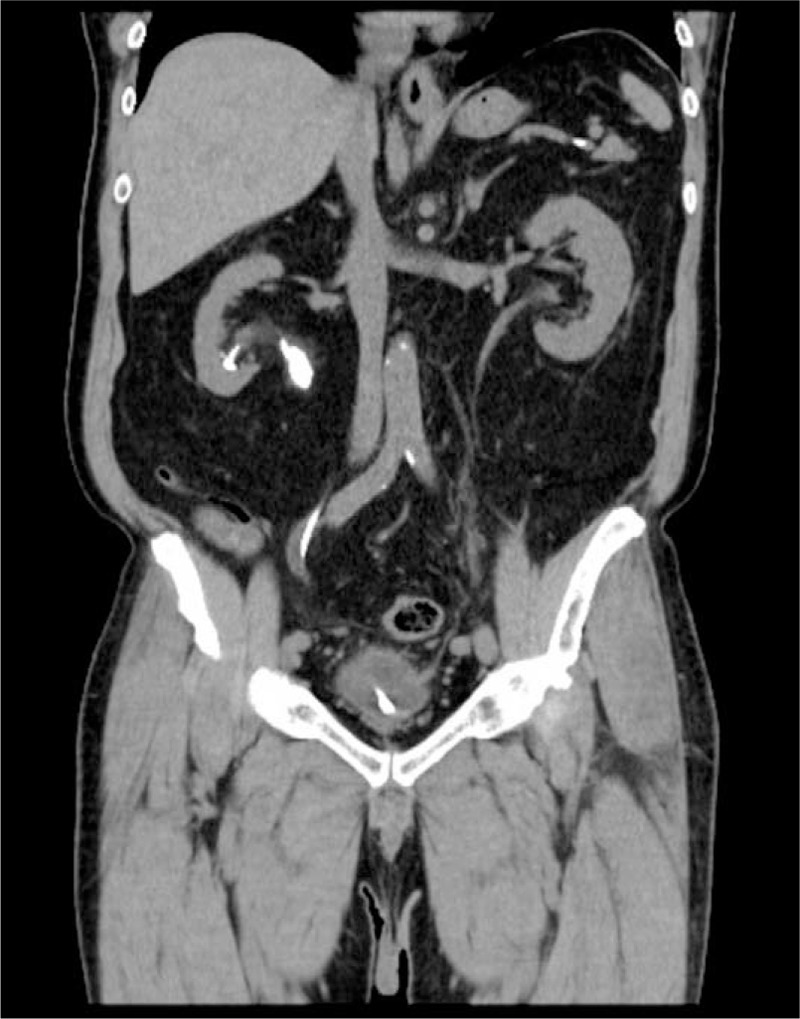
Preoperative computed tomography was arranged to identify the relative positions of the ureteropelvic junction stone, renal stones, and D-J stent. D-J = double-J catheter.

Laboratory studies, including tests for amylase, lipase, bilirubin, alanine, aspartate aminotransferases, and urine analysis were all within normal ranges. Computed tomography was performed to identify the relative positions of the UPJ stone, renal stones, and D-J stent to assess the pre-operative condition (Fig. 2).

**Figure 3:**
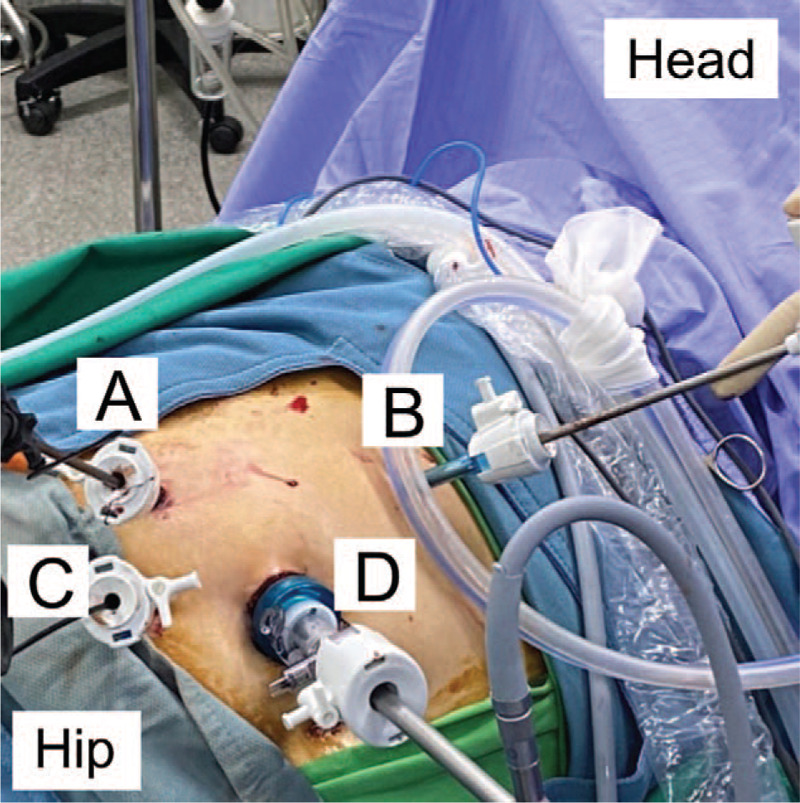
The laparoscopic port location and relative positions between ports. (A) working port; (B) working port; (C) fURS port; and (D) camera port. fURS = retrograde flexible ureteroscopy.

**Figure 4:**
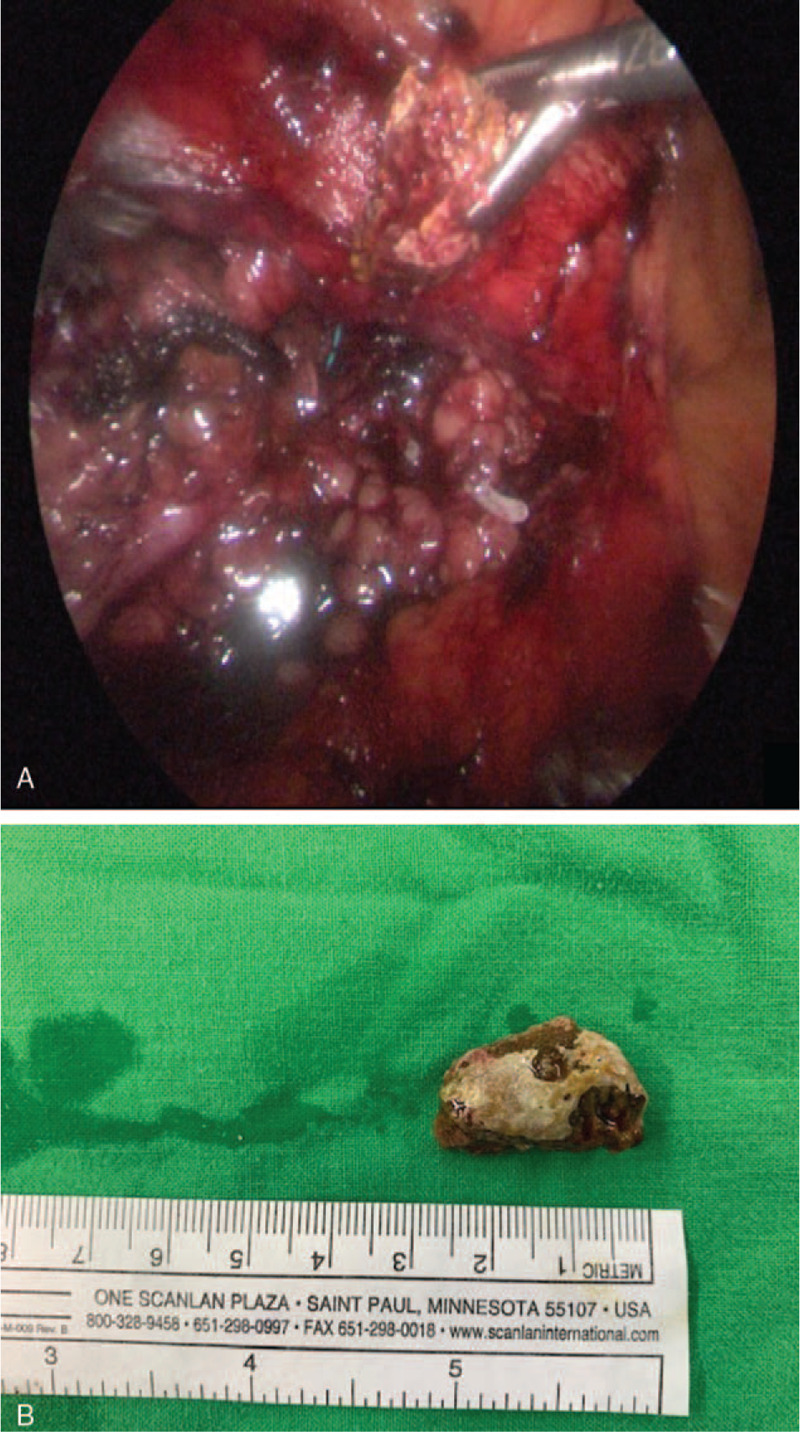
(A) Removal of the huge ureteral stone using a stone grasper during the course of laparoscopic ureterolithotomy. (B) En-bloc removal of the ureteral stone which was 2.8 cm in size.

**Figure 5:**
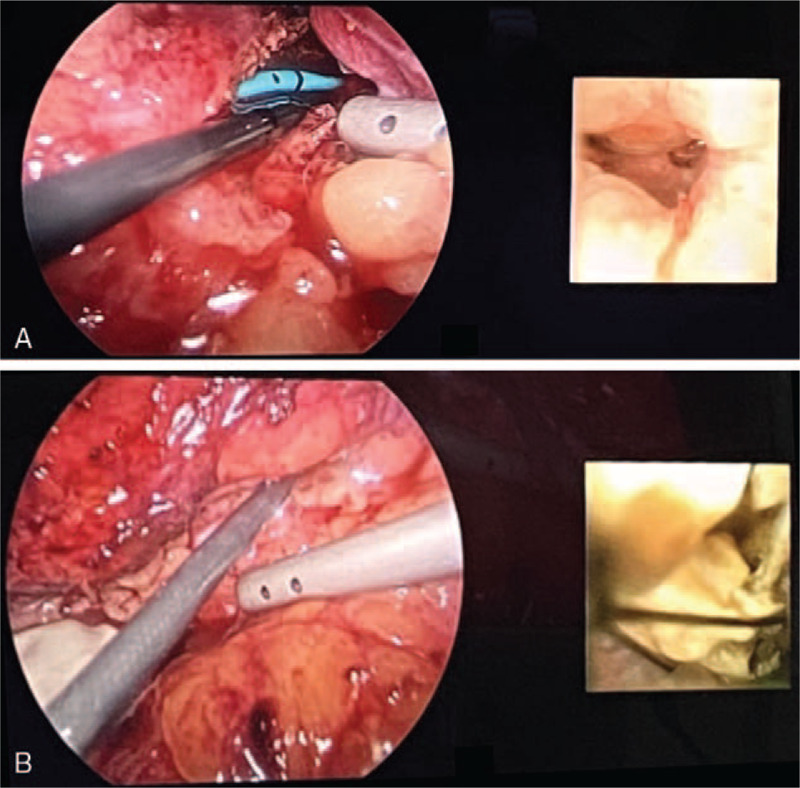
(A) Vision system concurrently provided a laparoscopic view and fURS intrarenal view, which was inserted through the ureterostomy opening. (B) The renal stones were identified under direct vision in fURS, and the stone was removed efficiently with a stone basket. fURS = retrograde flexible ureteroscopy.

We placed the patient in the left decubitus position with his right flank upward, and the procedure was performed through 4 ports (Fig. 3). An 11-mm trocar inserted 3 cm lateral to the umbilicus was used as the camera port using an open method. A 5-mm trocar (first working port) was placed in the subcostal area around the midclavicular line, and another 5-mm trocar (second working port) was placed 6 cm lateral to the camera port over the anterior axillary line. The third working port was a 5-mm assistant port over the midclavicular line 8 cm beneath the second working port. After reflection of the colon, the ureter was identified and mobilized, and the stone was located and extracted through a vertical ureterostomy. Using a stone grasper, the stone was extracted through the camera port (Fig. 4A and B). We then performed LA-RIRS after ureterolithotomy. We inserted a flexible ureteroscope through the third working port to perform a ureterostomy, and suctioned overflow normal saline from the outlet of the ureterostomy via the first port (Fig. 5A). Lower calyx stones were found and removed using a basket (Fig. 5B). The ureterostomy was closed as 2 layers with interrupted 4.0 Vicryl sutures to close the ureterostomy incision along the D-J stent. A 7-mm Jackson–Pratt drain was inserted through the second port. The total operation time from skin to skin was 1 hour and 27 minutes.

No complications were noted after the surgery, and the Jackson–Pratt drain was removed 3 days after the operation. The total hospital course was about 5 days.

**Figure 6:**
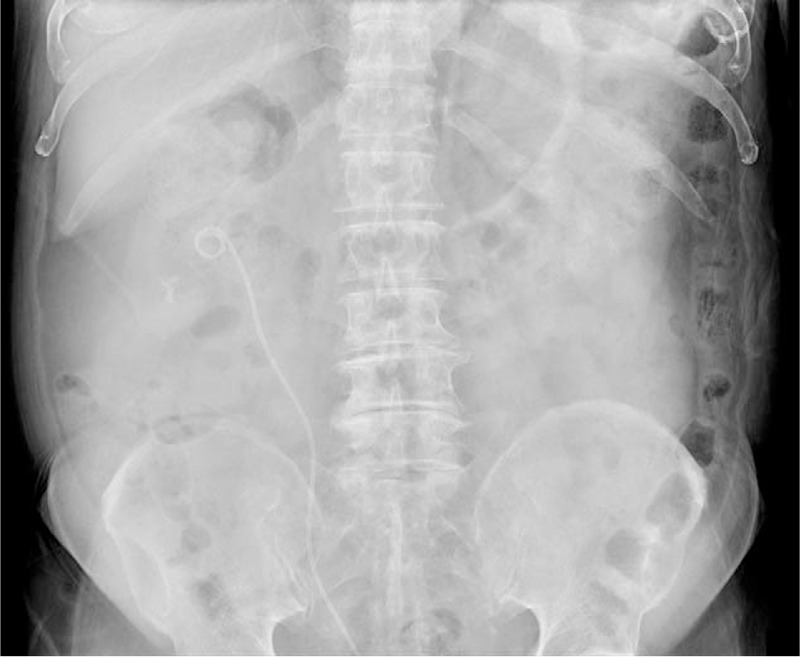
Postoperative kidney, ureter, and bladder radiograph showed no residual stones.

A follow-up kidney, ureter, and bladder radiograph (Fig. 6) were arranged 2 months after the operation, and no residual stones above 2 mm were found. Laboratory data measured during the same clinic visit showed a decline in creatinine from 1.63 to 1.1 mg/dL. The D-J stent was removed through cystoscopy after a radiograph in outpatient surgery. According to the Clavien Dindo classification, only a low grade II complication of a urinary tract infection was noted, which did not cause sepsis, and was treated with antibiotics.^[14]^ No high-grade complications occurred. A follow-up renal echo was performed 1 month after the D-J stent had been removed, which showed no obstructive nephropathy or significant residual renal calculi above 2 mm.

He is currently receiving regular follow-up of renal function, kidney, ureter, and bladder radiography, and renal echo every 6 months to 1 year.

## 3 Discussion

Urolithiasis frequently causes renal colic, which can lead to obstructive uropathy. Concurrent kidney and huge ureteral stones are always complicated and a clinical challenge in most situations.

Extracorporeal SWL is a less-invasive method that can be performed on an outpatient basis. The success rates of SWL for proximal ureteral stones vary widely, and the procedure is more complicated when renal calculi are involved.^[15,16]^ Factors that affect success rates include the patient's body mass index, stone diameter, degree of hydronephrosis, stone attenuation value, and SWL system.^[4,5,17]^ The rate of treatment success ranges from 49% to 96%.^[15,16]^ Moreover, additional treatments are needed in 43% of patients who undergo SWL due to ureteral obstruction caused by movement of smashed stones.^[22,23]^

PCNL is also a good alternative to manage large impacted upper ureteral stones.^[2–6,17]^ Moreover, it is possible to treat concurrent renal stones in the same session.^[4,6,17]^ The success rate of PCNL to treat upper ureteral stones >1.5 cm ranges from 85% to 100%.^[17]^ In addition, transfusion has been reported to be required in 2% to 5% of patients, although arterial embolization is rarely required.^[6,17]^ Recently, a decrease in the diameter of access tracts combined with fURS has expanded the role of PCNL.^[1]^

Improvements in endourological equipment have led to the widespread adoption of retrograde intrarenal surgery, which has a good stone clearance rate.^[1,2,18]^ With the recent development of smaller caliber flexible ureteroscopes and intracorporeal lithotripters, the success rate in treating renal stones has greatly increased.^[18]^ However, there are many limitations with retrograde intra-renal surgery.^[1,2,4]^

An ureterolithotomy is a minimally invasive modality that can be done under laparoscopic guidance. In 1992, Raboy et al first performed transperitoneal LUL.^[19]^ Its success rate is similar to open surgery with a relatively lower morbidity rate.^[19,20]^ According to the European Association guidelines on urolithiasis, large impacted ureteral stones, failure of minimally invasive procedures, different operative requirements for a concurrent indication, and technological deficiencies are indications for LUL.

In our case, LUL followed by LA-RIRS to extract the renal stones using a stone basket avoided a prolonged operative period and shortened the postoperative course. Compared to retrograde intrarenal surgery for the management of concurrent renal and huge ureteral stones, a higher stone-free rate, lower retreatment rate, and shorter operative time can be expected with LUL followed by LA-RIRS than with RIRS. More importantly, en-bloc removal of renal and ureteral calculi can avoid stone formation through a free particle mechanism, as reported by Vermeulen et al.^[21]^

In conclusion, LUL followed by LA-RIRS for renal stone extraction using a stone basket is a safe and feasible technique. It can be a treatment option for patients with large upper ureteral stones accompanied by renal stones who are indicated for LUL.

## 4 Ethical review

As this case report used only de-identified patient data and published data from the literature, no approval from our institutional review board (at China Medical University Hospital) was required.

## Acknowledgments

The authors would like to thank China Medical University Hospital for providing the opportunity to conduct this study.

## Author contributions

**Conceptualization:** Sheng-Feng Chou, Po-Fan Hsieh.

**Formal analysis:** Sheng-Feng Chou.

**Funding acquisition:** Chi-Ping Huang.

**Investigation:** Sheng-Feng Chou.

**Resources:** Po-Fan Hsieh.

**Software:** Wei-Ching Lin.

**Supervision:** Po-Fan Hsieh, Chi-Ping Huang.

**Validation:** Wei-Ching Lin.

**Visualization:** Wei-Ching Lin.

**Writing – original draft:** Sheng-Feng Chou.

**Writing – review & editing:** Po-Fan Hsieh.
